# Edge effect pinning in mesoscopic superconducting strips with non-uniform distribution of defects

**DOI:** 10.1038/s41598-018-36285-4

**Published:** 2019-01-18

**Authors:** Gregory J. Kimmel, Andreas Glatz, Valerii M. Vinokur, Ivan A. Sadovskyy

**Affiliations:** 1 Materials Science Division, Argonne National Laboratory, 9700 S Cass Av, Lemont, IL 60439 USA; 2 Department of Engineering Sciences and Applied Mathematics, Northwestern University, 633 Clark St, Evanston, IL 60208 USA; 3 Department of Physics, Northern Illinois University, DeKalb, IL 60115 USA; 4 Computation Institute, University of Chicago, 5735 S Ellis Av, Chicago, IL 60637 USA

## Abstract

Transport characteristics of nano-sized superconducting strips and bridges are determined by an intricate interplay of surface and bulk pinning. In the limiting case of a very narrow bridge, the critical current is mostly defined by its surface barrier, while in the opposite case of very wide strips it is dominated by its bulk pinning properties. Here we present a detailed study of the intermediate regime, where the critical current is determined, both, by randomly placed pinning centres and by the Bean-Livingston barrier at the edge of the superconducting strip in an external magnetic field. We use the time-dependent Ginzburg-Landau equations to describe the vortex dynamics and current distribution in the critical regime. Our studies reveal that while the bulk defects arrest vortex motion away from the edges, defects in their close vicinity promote vortex penetration, thus suppressing the critical current. We determine the spatial distribution of the defects optimizing the critical current and find that it is in general non-uniform and asymmetric: the barrier at the vortex-exit edge influence the critical current much stronger than the vortex-entrance edge. Furthermore, this optimized defect distribution has a more than 30% higher critical current density than a homogeneously disorder superconducting film.

## Introduction

Immobilizing magnetic vortices and thus preventing dissipation under applied currents is one of the major objectives for realizing applications of type-II superconductivity^1–4^. Typically, this vortex pinning is achieved by introducing structural inhomogeneities in the bulk of the material. Recently, it has been recognized that geometric pinning utilizing surface and geometrical barriers for controlling the entrance or exit of vortices in and out of mesoscopic superconductors and superconducting strips can be extremely efficient^5–12^. Appreciable enhancement of superconducting parameters in strips was recently observed experimentally and explained in terms of surface (edge) superconductivity^13,14^. One could conclude from these experiments that surfaces may provide one of the most important pinning mechanisms in strips and mesoscopic systems^15–17^. At the same time, it was observed that the introduction of point-like or cylindrical defects near the surface can be detrimental to the effectiveness of surface barriers^18,19^ since they promote easier vortex penetration across the surface^20^. Hence the effect of structural disorder is two-fold: it arrests the vortex dynamics in the bulk, but ‘contaminates’ surface pinning^21–24^. Both effects are important in an intermediate width regime where each mechanism contributes to the critical current, which is the largest possible applied current at which magnetic vortices are immobile.

In the case of narrow strips with widths on the order of the superconducting coherence length, the critical current is mostly defined by its surface barrier and phase slips across the strip are important^25,26^, while for very wide strips, the critical current is dominated by its bulk pinning properties. This sets the quest for optimizing artificially manufactured disorder in geometrically restricted systems to take advantage of a potentially constructive interplay of bulk and surface pinning mechanisms.

The present article addresses this problem. To this end, we design an approach allowing us to optimize the concentration and spatial distribution of the bulk point defects in order to achieve the maximum possible critical current taking into account the interplay between the surface barrier blocking penetration of vortices into a superconductor and bulk defects arresting the vortex motion in the interior of the sample. We consider experimentally important systems: superconducting wires having the shape of tapes with widths on the order of a few tens of the superconducting coherence length^3^. In order to calculate the critical current for a given arrangement of pins (pinscape), we use a solver for the time-dependent Ginzburg-Landau (TDGL) equation for type-II superconductors^27^. This approach describes the vortex dynamics sufficiently well in superconductors near the vicinity of the critical temperature and is capable of reproducing experimental critical currents for a given pinscape^28–31^.

## Model

We consider a two-dimensional superconducting strip, infinite in the *x* direction and a finite width *W*, which is appreciably larger than the superconducting coherence length, *ξ*, but less than the London penetration depth, *λ*. The edges at *y* = 0 and *y* = *W* set the positions of the surface barriers. Bulk defects are introduced by spatial modulation of the transition temperature, *T*_c_(**r**). To evaluate the critical current for the system, we use the TDGL equation, which simulates the dynamic behaviour of the complex superconducting order parameter *ψ* = *ψ*(**r**, *t*):

1 $$({\partial }_{t}+i\mu )\psi =\varepsilon ({\bf{r}})\psi -|\psi {|}^{2}\psi +{(\nabla -i{\bf{A}})}^{2}\psi +\zeta ({\bf{r}},t).$$

Here *μ* = *μ*(**r**, *t*) is the scalar potential, **A** is the vector potential generating the external magnetic field ${\bf{B}}=\nabla \times {\bf{A}}$, and *ζ*(**r**, *t*) is a temperature-dependent *δ*-correlated Langevin thermal noise term. The unit of length is defined by the superconducting coherence length *ξ* = *ξ*(*T*) at a given temperature *T* and the unit of the magnetic field is the upper critical field *H*_c2_ = *H*_c2_(*T*). Defects in the bulk are realized through the parameter *ε*(**r**) = [*T*_c_(**r**) − *T*]/[*T*_c,bulk_ − *T*], where *T*_c,bulk_ is the transition temperature for the clean sample. We solve the TDGL equation in the infinite-*λ* limit, allowing us to use the gauge **A** = (−*B*_*z*_, 0, 0)*y* for the vector potential.

**Figure 1:**
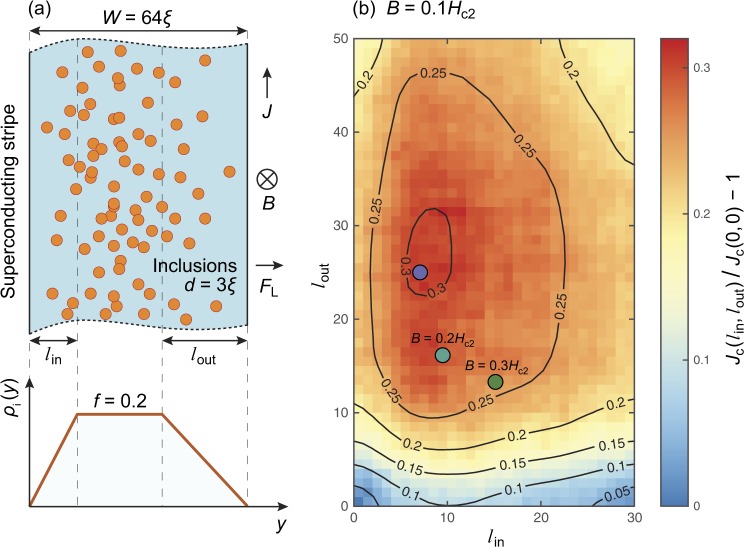
(**a**) Two-dimensional superconducting strip of width *W* = 64*ξ* with non-homogeneous inclusion distribution. The current *J* is applied vertically (along the *x*-axis), the magnetic field *B* is perpendicular to the figure plane, and the resulting Lorentz force *F*_L_ acts to the right (along the *y*-axis). The sample has a length of *L* = 1024*ξ* with quasi-periodic boundary conditions in the *x* direction; in the *y* direction, we have open boundary conditions, i.e., superconductor-vacuum surfaces. The strip contains (uncorrelated) randomly placed circular inclusions of diameter *d* = 3*ξ*. The density of these inclusions depends on *y*: in the middle of the sample, the volume fraction occupied by inclusions is *f* = 0.2, which corresponds approximately to conditions for the maximum possible critical current density in bulk samples. The density of the inclusion *ρ*_i_(*y*) decreases linearly near the sample boundaries (see bottom plot): within a region of width *l*_in_ at the boundary where vortices enter the sample and *l*_out_ at the boundary where vortices leave the sample. (**b**) The critical current *J*_c_ as a function of *l*_in_ and *l*_out_ normalized by *J*_c_(0, 0) at applied magnetic field *B* = 0.1*H*_c2_. The critical current is increased by ~30% for finite *l*_in_ and *l*_out_ compared to the critical current from a homogeneous defect distribution (*l*_in_ = *l*_out_ = 0). The values of *l*_in_ and *l*_out_ corresponding to the maximum of the critical current *J*_c_(*l*_in_, *l*_out_) are shown by colored circles for *B* = 0.1*H*_c2_, 0.2*H*_c2_, and 0.3*H*_c2_. The effect is asymmetric and depends on the direction of vortex motion. The maximum is indicated by a (blue) circle. Corresponding maxima for fields 0.2*H*_c2_ and 0.3*H*_c2_ are indicated by (cyan and green) circles, marked by the field value.

We solve Eq. (1) numerically by discretising the system on a regular grid with mesh size of half a coherence length and integration of time using an implicit massively parallel iterative solver, see ref.^27^ for implementation details. We consider the model system shown in Fig. 1(a), where the two-dimensional superconducting strip lies in the *xy* plane with quasi-periodic boundary conditions imposed in *x* direction and open boundary conditions in *y* direction (i.e., the *y* component of the current has to obey *J*_*y*_ = 0 at these boundaries corresponding to a superconductor-vacuum surface). The magnetic field *B* is applied in *z* direction and the external current *J* is applied in the *x* direction. In this case, the Lorentz force drives vortices in +*y* direction (i.e., vortices enter the domain from *y* = 0 and exit at *y* = *W*).

The current density,

2 $${\bf{J}}=\frac{3\sqrt{3}}{2}\{{\rm{Im}}[{\psi }^{\ast }(\nabla -i{\bf{A}})\psi ]-\nabla \mu \}$$

is measured in units of the *depairing current J*_dp_ = *J*_dp_(*T*). *J*_dp_ is the current at which the superconducting order parameter is suppressed to zero, or Cooper pairs are not stable anymore, i.e., superconductivity is completely destroyed.

The magnitude of the critical current in the presence of an external magnetic field is controlled by inclusion patterns, which are small non-superconducting islands immersed in the superconducting matrix. We tune the inclusion size (typically a few *ξ*) and their spatial distribution.

To determine the magnitude of the critical current, we use a finite-electrical-field criterion. Specifically, we chose a certain small external electric field, ${E}_{{\rm{c}}}={10}^{-4}\mathrm{(3}\sqrt{3}/\mathrm{2)}{J}_{{\rm{dp}}}/\sigma$, where *σ* is the normal conductivity, and adjust the external current, *J*, to keep this electrical-field criterion on average. The time-averaged value of the external current in the steady state gives the critical current, *J*_c_ = 〈*J*〉. We start with the two limiting situations: a clean strip and bulk superconductor with defects.

### Clean strip

**Figure 2:**
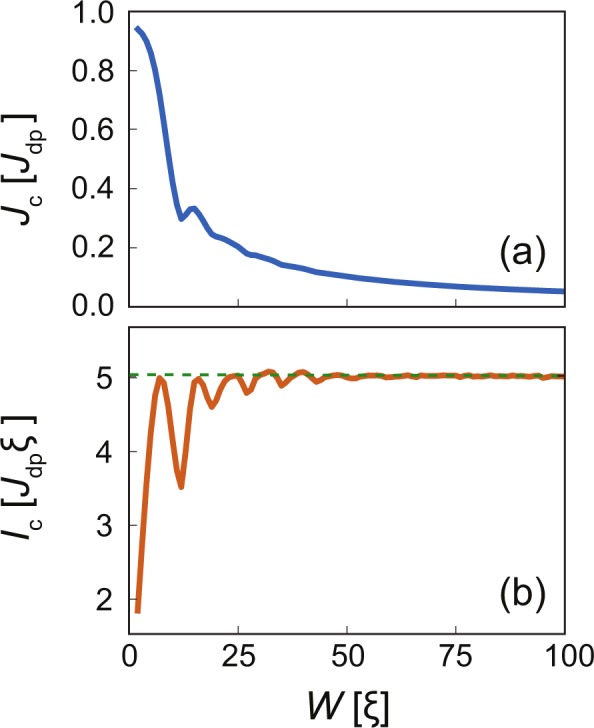
(**a**) Critical current density *J*_c_ and (**b**) critical current *I*_c_ = *J*_c_*W* as a function of width *W* of the ideal superconducting strip containing no inclusions in magnetic field *B* = 0.1*H*_c2_ applied perpendicular to the strip. The critical current is defined by strip boundaries only and saturates at *I*_c_ ≈ 5*J*_dp_*ξ* (green dashed line) for $W\gtrsim 64\xi$ due to the absence of pinning potentials in the bulk. Certain vortex configurations with few commensurate vortex rows (in particular the 4 and 5 row configuration) are very stable due to geometrical pinning and can have larger critical currents than the saturation value, see ref.^10^. Artifacts from the constant voltage criterion, used to determine the critical current, for wide clean strips are removed, see text.

The pinning force in this case is defined by edges at *y* = 0 and *y* = *W* with open (no-current) boundary conditions. These boundaries produce the Bean-Livingston barrier^18,19,32–35^ and arrange vortices in ‘rows’ along the current direction^10^. The number of rows depends on the width of the strip *W* and on the applied magnetic field *B*. At fixed magnetic field, the most stable configurations are achieved under commensurability conditions. Therefore upon changing the width, the number of the stable rows varies as well, leading to oscillations in the critical current density *J*_c_(*W*), which are more pronounced in the total critical current *I*_c_(*W*) = *J*_c_(*W*)*W* as shown in Fig. 2(a,b), respectively. The maxima are realized when the system can accommodate the number of vortices corresponding to the applied field and minima when the system is in between two stable vortex lattice configurations. These oscillations can be best observed for the first few vortex rows. For $W\gg 1$, the critical current *I*_c_ saturates at some certain value defined by the depinning forces of the two barriers and depends on the magnetic field. Note, that certain commensurate vortex configurations are very stable (in particular for 4 or 5 rows), such that the critical current for these configurations can be even larger than the saturation value. We remark that the method to determine the critical current described above is independent of *W*, which leads to small linear increase in the critical current with the width of the system as the critical current density saturates when the free-flow voltage (the free-flow regime is the regime of linear current-voltage behaviour where vortices are not pinned anymore) is equal to the chosen electric field cutoff (which determines the slope of increase). This artificial increase becomes recognizable for very wide systems and is therefore subtracted from the critical current in Fig. 2(b).

### Bulk superconductor

In this case, the critical current associated with pinning vortices at non-superconducting defects depends on the defect properties (shape, size, concentration) and on the field strength (vortex density). In a three-dimensional (3D) bulk type-II superconductor containing spherical particles and for a wide range of fixed applied magnetic fields, 0.02*H*_c2_ < *B* < 0.2*H*_c2_, the optimal critical current is achieved for particle diameters *d* ranging from 2.5*ξ* to 4.5*ξ* and 15–20% volume fraction occupied by particles^36^. For large inclusions of fixed diameter $d\geqslant 3\xi$, the field dependence of the critical current has shown peculiar peaks, associated with the inclusion’s occupancy by multiple vortices^37,38^. Similar results are observed in regular and random pinning configurations of circular (cylindrical) defects in two-dimensional (3D) systems^31,39^. Note that a 2D system with circular defects is comparable to a 3D system with columnar rather than spherical defects, see below.

### General case

Now, we consider geometrically confined 2D systems with circular defects. We design the pinning configuration within our model system with finite *W* in the following way: (i) the density of the non-superconducting columnar defects far away from the edges is the same as in the bulk case corresponding to the maximum possible critical current; (ii) the density of non-superconducting defects near edges is linearly modulated towards the edges. We define the volume fraction *ρ*_i_(*y*) occupied by defects of the same diameter *d* as a function of *y* which is given by

3 $${\rho }_{{\rm{i}}}(y)=\{\begin{array}{cc}\frac{<mml:mpadded xmlns:xlink="http://www.w3.org/1999/xlink" width="0" height="8.6pt" depth="3pt"/>{\textstyle y}}{<mml:mpadded xmlns:xlink="http://www.w3.org/1999/xlink" width="0" height="8.6pt" depth="3pt"/>{\textstyle {l}_{{\rm{i}}{\rm{n}}}}}\,f+\frac{<mml:mpadded xmlns:xlink="http://www.w3.org/1999/xlink" width="0" height="8.6pt" depth="3pt"/>{\textstyle {l}_{{\rm{i}}{\rm{n}}}-y}}{<mml:mpadded xmlns:xlink="http://www.w3.org/1999/xlink" width="0" height="8.6pt" depth="3pt"/>{\textstyle {l}_{{\rm{i}}{\rm{n}}}}}\,{f}_{{\rm{i}}{\rm{n}}}, & y < {l}_{{\rm{i}}{\rm{n}}}\\ f, & {l}_{{\rm{i}}{\rm{n}}}\leqslant y\leqslant W-{l}_{{\rm{o}}{\rm{u}}{\rm{t}}}\\ \frac{<mml:mpadded xmlns:xlink="http://www.w3.org/1999/xlink" width="0" height="8.6pt" depth="3pt"/>{\textstyle W-y}}{<mml:mpadded xmlns:xlink="http://www.w3.org/1999/xlink" width="0" height="8.6pt" depth="3pt"/>{\textstyle {l}_{{\rm{o}}{\rm{u}}{\rm{t}}}}}\,f+\frac{<mml:mpadded xmlns:xlink="http://www.w3.org/1999/xlink" width="0" height="8.6pt" depth="3pt"/>{\textstyle {l}_{{\rm{o}}{\rm{u}}{\rm{t}}}+y-W}}{<mml:mpadded xmlns:xlink="http://www.w3.org/1999/xlink" width="0" height="8.6pt" depth="3pt"/>{\textstyle {l}_{{\rm{o}}{\rm{u}}{\rm{t}}}}}\,{f}_{{\rm{o}}{\rm{u}}{\rm{t}}}, & y > W-{l}_{{\rm{o}}{\rm{u}}{\rm{t}}}.\end{array}$$

In particular, the volume fraction of the defects changes linearly from *f*_in_ to its bulk value *f* at the distance *l*_in_ from the edge *y* = 0 where vortices enter the sample. On the opposite side of the sample *ρ*_i_(*y*) changes from *f* to *f*_out_ at distance *l*_out_.

## Results

The surface barrier at the superconductor edges prevent vortices from entering and exiting the superconductor. As mentioned in the introduction, non-superconducting defects located at edges or in the vicinity of edges effectively reduce the Bean-Livingston barrier by creating weak spots for vortex penetration^22^. We study the interplay between the surface barrier and defect distribution profile *ρ*_i_(*y*) by investigating the dependence of the critical current density, *J*_c_, on the parameters *f*, *f*_in_, *f*_out_, *l*_in_, *l*_out_, *d*, in a fixed magnetic field *B* and fixed sample width $W\gg {l}_{{\rm{in}}}$, *l*_out_. Therefore, we start our numerical investigation with initial investigations of the full 6D optimization problem

4 $${{\bf{p}}}^{{\rm{o}}{\rm{p}}{\rm{t}}}=\arg \,\mathop{max}\limits_{{\bf{p}}}\,{J}_{{\rm{c}}}({\bf{p}})$$

with control parameter set **p** = {*f*, *f*_in_, *f*_out_, *l*_in_, *l*_out_, *d*} for different fixed magnetic fields using a particle swarm optimization routine^39^. The resulting optimal parameter set **p**^opt^ corresponds to the maximum critical current density *J*_c_(**p**^opt^). These initial studies revealed that for the range of applied magnetic fields investigated in this paper, the optimal concentrations of the defects near the entrance and exit boundaries were zero, ${f}_{{\rm{in}}}^{{\rm{opt}}}={f}_{{\rm{out}}}^{{\rm{opt}}}=0$. This allows us to simplify the initial model density profile (3) to

5 $${\rho }_{{\rm{i}}}(y)=\{\begin{array}{cc}\frac{<mml:mpadded xmlns:xlink="http://www.w3.org/1999/xlink" width="0" height="8.6pt" depth="3pt"/>{\textstyle y}}{<mml:mpadded xmlns:xlink="http://www.w3.org/1999/xlink" width="0" height="8.6pt" depth="3pt"/>{\textstyle {l}_{{\rm{i}}{\rm{n}}}}}\,f, & y < {l}_{{\rm{i}}{\rm{n}}},\\ f, & {l}_{{\rm{i}}{\rm{n}}}\le y\le W-{l}_{{\rm{o}}{\rm{u}}{\rm{t}}},\\ \frac{<mml:mpadded xmlns:xlink="http://www.w3.org/1999/xlink" width="0" height="8.6pt" depth="3pt"/>{\textstyle W-y}}{<mml:mpadded xmlns:xlink="http://www.w3.org/1999/xlink" width="0" height="8.6pt" depth="3pt"/>{\textstyle {l}_{{\rm{o}}{\rm{u}}{\rm{t}}}}}\,f, & y > W-{l}_{out}\end{array}$$

shown in Fig. 1(a), leaving four parameters to optimize.

The optimal particle diameter *d*^opt^ decreases with the applied filed *B* and ${d}^{{\rm{opt}}}\approx 3\xi$ for *B* = 0.1*H*_c2_. This result is different from that in the 3D case for spherical particles, which has an optimal diameter of *d*^opt^ ≈ 4*ξ* for the same field. This discrepancy in the result is due to the fact that the 2D circular defects we model correspond to columnar defects in 3D samples. It was found earlier that the optimal diameter of columnar defects is smaller than the optimal diameter of spherical defects by approximately one coherence length *ξ*. Since the optimal volume fraction *f* = 0.2 and diameter of defects *d* = 3*ξ* in both cases are similar^39^, we keep them constant in the following analysis, making the optimization problem manageable and effectively a two parameter optimization problem.

Figure 1(b) demonstrates the dependency of the critical current on the distance with reduced defect density at the entrance *l*_in_ and exit *l*_out_ of vortices for a sample of width *W* = 64*ξ*. One can see that the effect is far from symmetric. Figure 1(b) at *B* = 0.1*H*_c2_ shows that the critical current has a maximum of *J*_c_(*l*_in_, *l*_out_) ≈ 1.3*J*_c_(0, 0) at *l*_in_ ≈ 10*ξ* and *l*_out_ ≈ 30*ξ*. The *J*_c_(*l*_in_, *l*_out_) maxima are indicated by colored circles for *B* = 0.1*H*_c2_, 0.2*H*_c2_, and 0.3*H*_c2_. The dependence presented in Fig. 1(b) is a result of the interplay between pinning on inclusions and the Bean-Livingston barrier near the superconducting strip edge. For larger external fields the optimal entrance and exit regions become more symmetric as see by the maxima of *J*_c_ for *B* = 0.2*H*_c2_, 0.2*H*_c2_, indicated by circles in Fig. 1(b). In particular *l*_out_ becomes smaller with increasing *B*, approaching *l*_in_, and the overall critical current peak becomes wider, i.e., the system is less sensitive to *l*_in_ and *l*_out_ at larger *B*.

**Figure 3:**
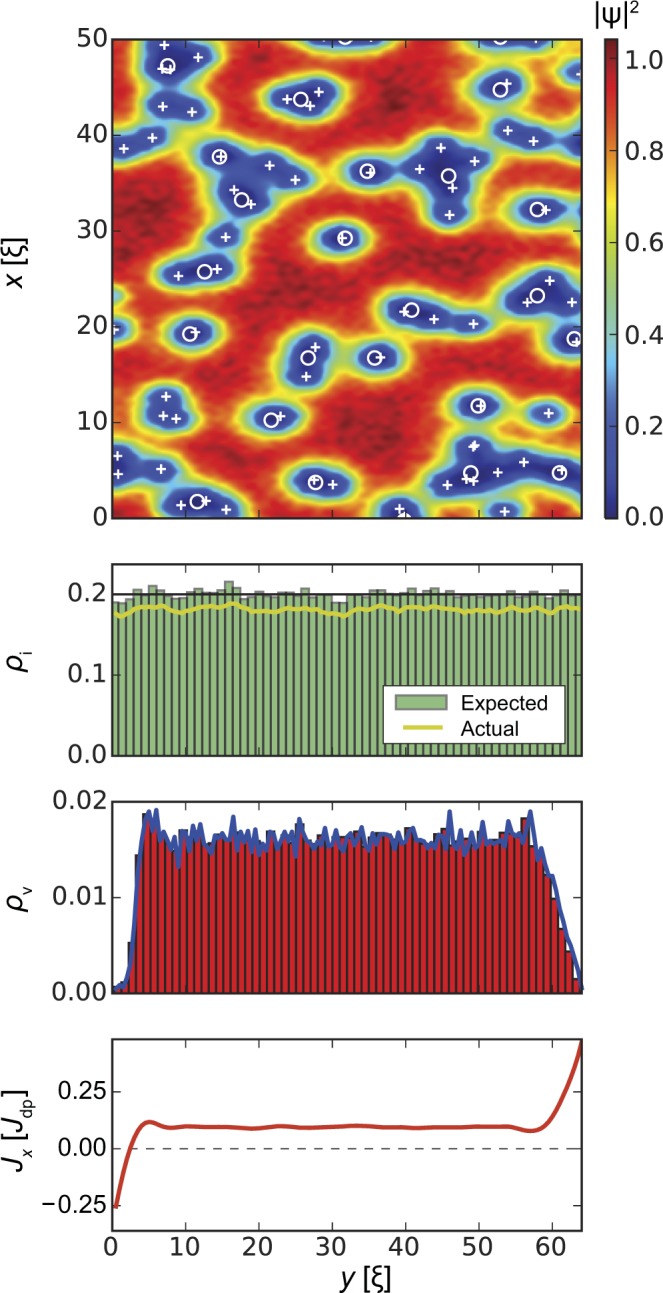
Strip with homogeneous distribution of inclusion density, *l*_in_ = *l*_out_ = 0, *ρ*_i_ = *f* = 0.2 in an applied magnetic field *B* = 0.1*H*_c2_. *Top panel* shows the squared absolute value of the order parameter |*ψ*(**r**)|^2^. Circles and crosses show inclusion and vortex positions, respectively. *Second panel* shows the distribution of the inclusions across the strip (*y* direction). The black line shows the ‘requested’ volume fraction *f* = 0.2, the green histogram shows the distribution of the centres of the inclusions, and the yellow line shows the actual volume fraction occupied by the generated defects. (the actual volume fraction is typically lower than the specified/requested one due to defect overlaps and fluctuations of finite random number sequences.) *Third panel* demonstrates the density *ρ*_v_ of vortices. *Bottom panel* shows the local current density *J*_*x*_(*y*). As expected, the edge screening currents at the surface are in opposite directions, while the small local minimum and maximum a few *ξ* away from the edge are related to an alignment of vortices at the interior surface barrier. The average critical current density is ${J}_{{\rm{c}}}^{{\rm{uniform}}}=0.108{J}_{{\rm{dp}}}$.

**Figure 4:**
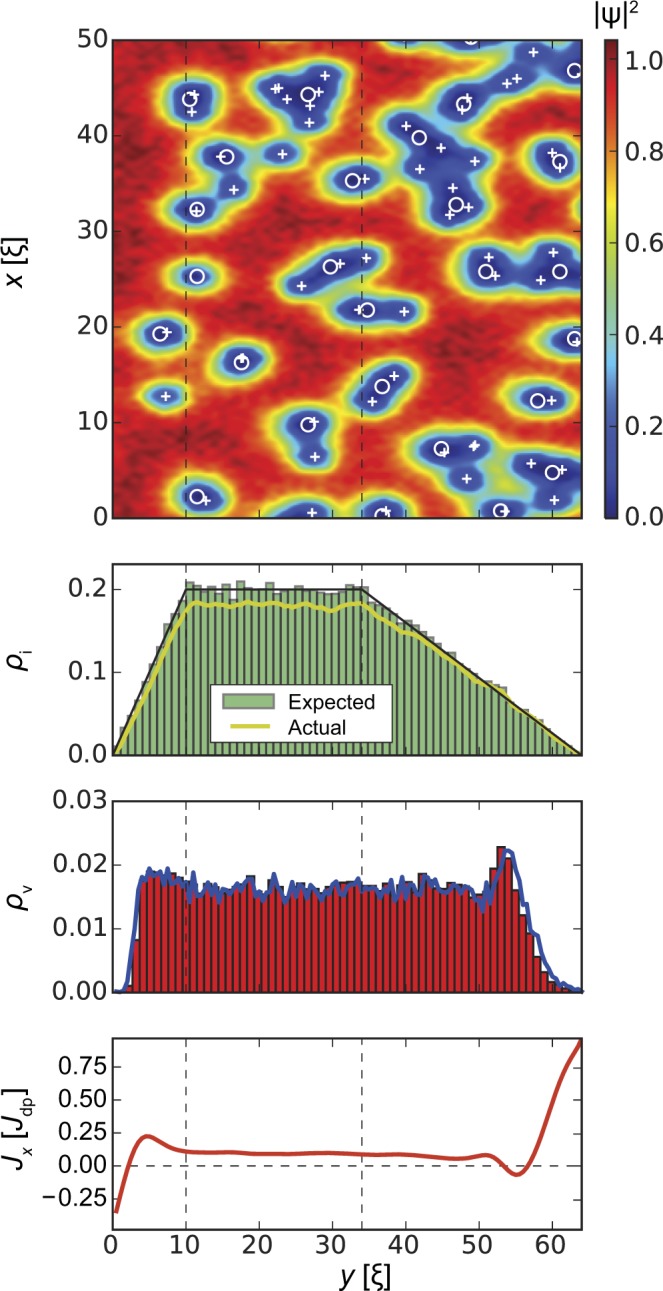
A strip with reduced inclusion density at both edges *l*_in_ = 10*ξ*, *l*_out_ = 30*ξ*, *f* = 0.2, and *B* = 0.1*H*_c2_. The average critical current ${J}_{{\rm{c}}}^{{\rm{both}}}=0.14{J}_{{\rm{dp}}}$ is 28% larger compared to Fig. 3. The *J*_*x*_(*y*) dependence has much more pronounced features near the edges. These oscillations in the current are generated by (free) vortex rows in the region of low inclusion density.

**Figure 5:**
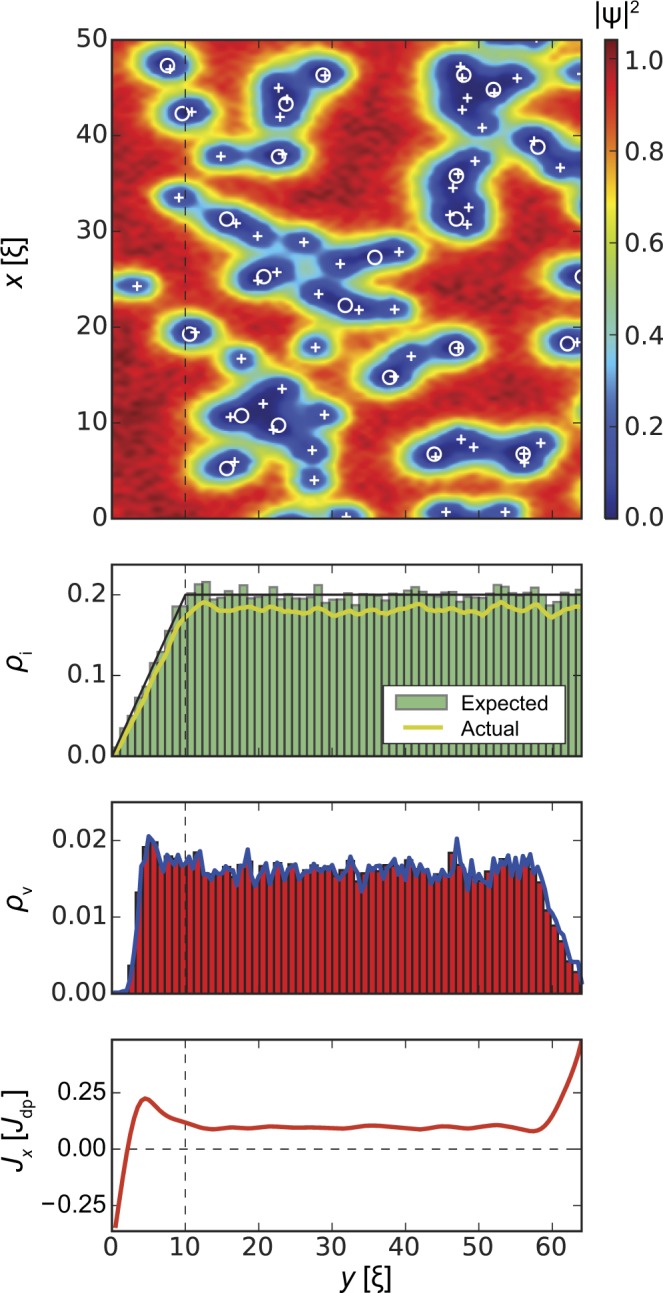
A strip with reduced inclusion density on the entrance side only, *l*_in_ = 10*ξ* and *l*_out_ = 0, has an average critical current density of ${J}_{{\rm{c}}}^{{\rm{in}}}=0.118{J}_{{\rm{dp}}}$ at applied magnetic field *B* = 0.1*H*_c2_.

**Figure 6:**
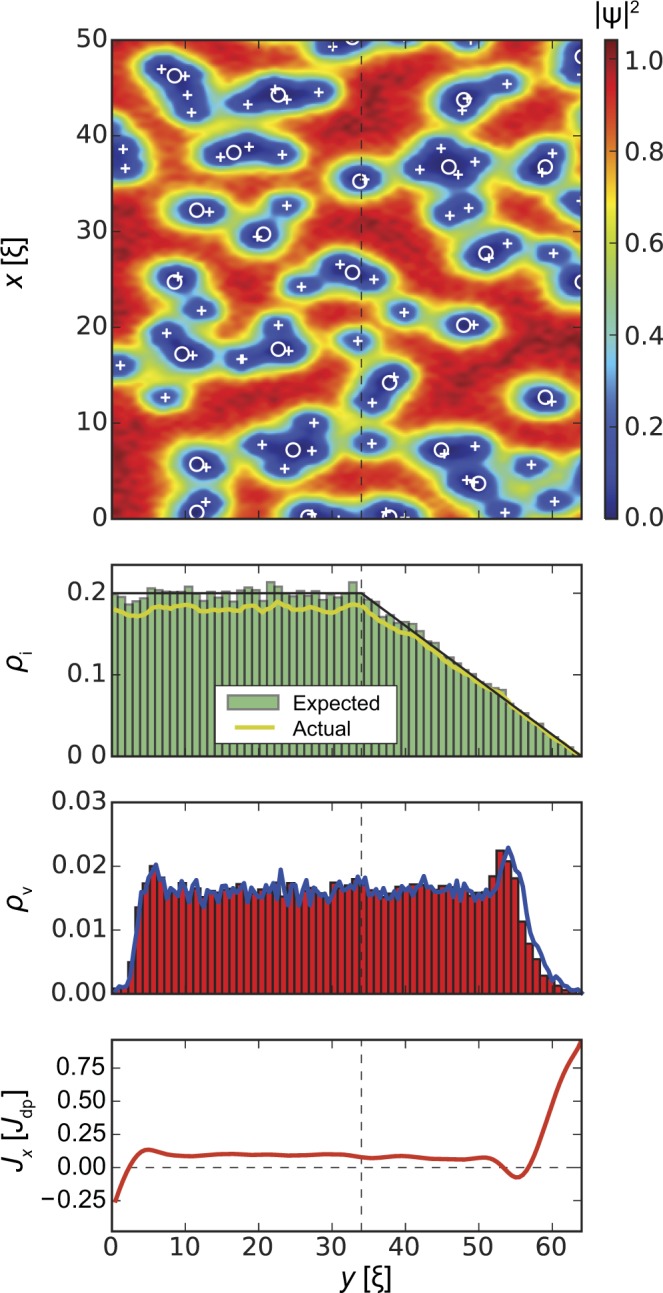
A strip with reduced inclusion density at the exit side only, *l*_in_ = 0 and *l*_out_ = 30*ξ*. The average critical current density is ${J}_{{\rm{c}}}^{{\rm{out}}}=0.131{J}_{{\rm{dp}}}$ at *B* = 0.1*H*_c2_.

In the following we will discuss this interplay in detail. Our results are summarized in Figs 3, 4, 5 and 6. All figures have the same format. *Top panels* show the squared absolute value of the order parameter |*ψ*(**r**)|^2^ in samples of width *W* = 64*ξ* (*y* direction) and length *L* = 1024*ξ* (*x* direction; quasi-periodic boundary conditions). White circles correspond to inclusions, white crosses indicate vortex positions. The presented order parameter configurations are for applied currents *J*_*x*_ = *J*_c_. *Second panels* show the distribution of defects along the *y* direction and averaged over the length of the strip (*x* direction). The black lines indicate the requested volume fraction *ρ*_i_(*y*) defined by Eq. (5) with *f* = 0.2 (i.e. 20% of the volume occupied by inclusions in the bulk, which corresponds to *B*_Φ_/*B* = 1.78 inclusions per vortex at *B* = 0.1*H*_c2_, where *B*_Φ_ = 8*f*/*d*^2^ is the matching field of the columnar pinning landscape defined by parameters *f* and *d*), the green histograms show the distribution of the centres of the inclusions, and the yellow lines show the actual volume fraction occupied by the generated defects. The latter value is somewhat lower than the requested value due to overlapping of inclusions and finite size effects, the real/actual volume fraction can be estimated as ${\rho }_{{\rm{i}}}^{\ast }(y)=1-\exp [\,-\,{\rho }_{{\rm{i}}}(y)]$. The requested bulk defect density corresponding to a volume fraction *f* = 0.2 has ${f}^{\ast }\approx 0.181$ real volume fraction. Inclusions overlapping effectively changes the matching field to ${B}_{{\rm{\Phi }}}^{\ast }=8{f}^{\ast }/{d}^{2}$ and number of inclusions per one vortex to ${B}_{{\rm{\Phi }}}^{\ast }/B=1.61$. *Third panels* demonstrate the density of the vortices *ρ*_v_(*y*) averaged over the length of the strip. In all cases, the vortex density tends to zero at *y* = 0 and *y* = *W* and remains roughly constant in the bulk of the superconductor. *Bottom panels* show the *x*-component of the local current density, *J*_*x*_(*y*), averaged over the length of the strip and are indicative of the edge currents and reflect the distribution of vortices.

Vortex and current density distributions for homogeneous inclusion density *ρ*_i_ = *f* = 0.2 for 0 < *y* < *W* (*l*_in_ = *l*_out_ = 0) are shown in Fig. 3. The position of vortices is strongly correlated with the particular placement of the inclusions, which makes the visual analysis rather complicated. The histograms of defects, vortices, and *x*-component of current averaged over the sample length *L* and 10 different realizations of defect distributions contain more useful information. The vortex density is approximately constant in the bulk. This density decreases to zero at ~5*ξ* away from both edges due to the Bean-Livingston barrier. Such a rapid gradient in vortex density produces large surface currents, which has a density on the order of the depairing current density *J*_dp_. The average critical current density is ${J}_{{\rm{c}}}^{{\rm{uniform}}}=0.108{J}_{{\rm{dp}}}$.

Figure 4 shows how the result changes when we reduce the inclusion density at both edges of the superconducting strip. We pick *l*_in_ = 10*ξ*, *l*_out_ = 30*ξ*, with the remaining volume fraction of inclusions in the bulk as *f* = 0.2 and applied magnetic field *B* = 0.1*H*_c2_. The chosen parameters are close to the maximum of *J*_c_(*l*_in_, *l*_out_) shown in Fig. 1(b). The critical current ${J}_{{\rm{c}}}^{{\rm{both}}}=0.14{J}_{{\rm{dp}}}$ represents a 30% increase compared to uniform inclusion density ${J}_{{\rm{c}}}^{{\rm{uniform}}}$. At the same time, the bulk critical current density (for ${l}_{{\rm{in}}}\lesssim y\lesssim L-{l}_{{\rm{out}}}$) remains approximately the same. This indicates that the critical current enhancement is mostly related to the defect distribution near the boundaries of the superconducting strip.

Comparing the vortex configuration in that case with that of the uniform inclusion density case, where the location of vortices is mostly random, we find that this *J*_c_ enhancement is produced by the formation of regular vortex row(s) in the regions with a reduced concentration of defects. Each vortex row can be interpreted as an additional potential barrier parallel to the edge repelling vortices. However, since current circulates around each vortex in the row, we can observe the local current flowing in the positive *x* direction to the right of vortex row and the current flowing in the negative *x* direction to the left of the vortex row. The value of this local current can be as high as the depairing current density, *J*_dp_. This current density can be observed at *y* = *W* in Fig. 4. The value of this current is somewhat lower in between rows due to cancellation of opposite screening currents from rows at the left and at the right. Overall, these regular (mostly unpinned) rows lead to oscillations of the average vortex density and subsequently the current density along the applied current direction. This effect is similar to the one observed in artificially manufactured vortex-flow channels in irradiated mesoscopic samples^40^.

Next we examine how the reduced inclusion density affects the superconducting strip edge where vortices enter and exit the sample separately. The results for the strip with reduced inclusion density at the entrance side only, *l*_in_ = 10*ξ* and *l*_out_ = 0, is presented in Fig. 5. This pinning landscape generates an average critical current density ${J}_{{\rm{c}}}^{{\rm{in}}}=0.118{J}_{{\rm{dp}}}$. One sees that ‘entrance’ and bulk parts of all histograms, $y\lesssim W/2$, coincides with the corresponding part of Fig. 4 and ‘exit’ and bulk parts $y\gtrsim W/2$ reproduces the same regions in Fig. 3. An analogous situation appears with reduced inclusion density at the exit side of the strip (Fig. 6), *l*_in_ = 0 and *l*_out_ = 30*ξ*. This configuration produces an average critical current density ${J}_{{\rm{c}}}^{{\rm{out}}}=0.131{J}_{{\rm{dp}}}$.

Naturally, values of ${J}_{{\rm{c}}}^{{\rm{in}}}$ and ${J}_{{\rm{c}}}^{{\rm{out}}}$ are in between the two critical current densities of the strip with uniform inclusion distribution and the strip with reduced inclusion density on both edges, i.e., ${J}_{{\rm{c}}}^{{\rm{uniform}}} < {J}_{{\rm{c}}}^{{\rm{in}}}$, ${J}_{{\rm{c}}}^{{\rm{out}}} < {J}_{{\rm{c}}}^{{\rm{both}}}$. The independence of the vortex and current configurations on the left and right edges can also be confirmed by comparing differences in the average (or total) critical current of the four configurations discussed above. In particular, ${J}_{{\rm{c}}}^{{\rm{both}}}+{J}_{{\rm{c}}}^{{\rm{uniform}}}={J}_{{\rm{c}}}^{{\rm{in}}}+{J}_{{\rm{c}}}^{{\rm{out}}}$ holds for all wide enough strips, $W\gtrsim {l}_{{\rm{in}}}+{l}_{{\rm{out}}}$.

Taking into account that (i) the chosen *l*_in_ = 10*ξ* and *l*_out_ = 30*ξ* correspond to the nearly largest critical current at the given magnetic field and (ii) entrance and exit edges act almost independently, we can say that the edge barrier at the entrance can generate additional critical current up to $\delta {I}_{{\rm{c}}}^{{\rm{in}}}=({J}_{{\rm{c}}}^{{\rm{in}}}-{J}_{{\rm{c}}}^{{\rm{uniform}}})W=0.51{J}_{{\rm{dp}}}\xi$, while the same addition at the exit edge $\delta {I}_{{\rm{c}}}^{{\rm{out}}}=({J}_{{\rm{c}}}^{{\rm{out}}}-{J}_{{\rm{c}}}^{{\rm{uniform}}})W=1.54{J}_{{\rm{dp}}}\xi$ is three times bigger. Note, that the clean strip with ideal boundaries (without any inclusions in the bulk) can generate a total critical current up to *I*_c_ ≈ 5.1*J*_dp_*ξ* at the same applied magnetic field, see Fig. 2(b).

**Figure 7:**
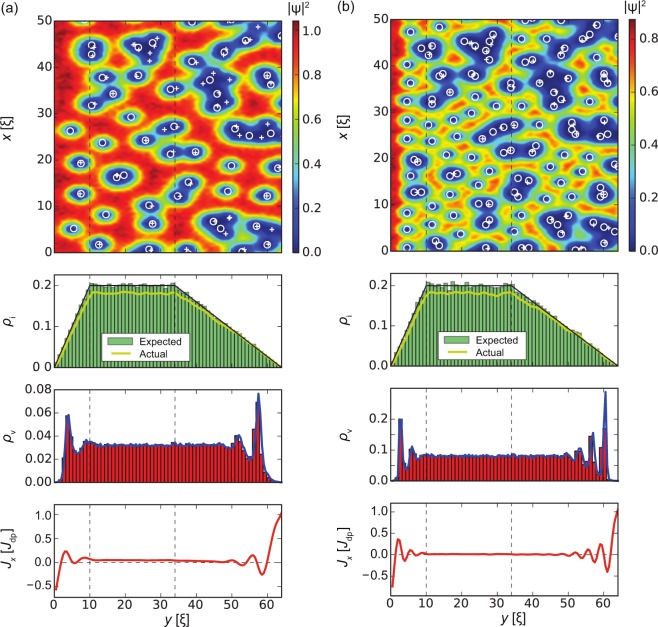
The same as in Fig. 4, but for higher magnetic fields. (**a**) Field *B* = 0.2*H*_c2_ produces an average critical current density *J*_c_ = 0.075*J*_dp_ and (**b**) field *B* = 0.5*H*_c2_ generates *J*_c_ = 0.026*J*_dp_. At higher fields, vortex rows are more dense. This leads to faster oscillations in vortex and local current densities *J*_*x*_(*y*). At the exit edge *J*_*x*_(*y*) reaches *J*_dp_.

Higher magnetic fields decreases the distance between neighbouring vortex rows and thus leads to higher frequency oscillations of vortex density and the *x*-component of current in regions with reduced inclusion density as shown in Fig. 7. A magnetic field *B* = 0.2*H*_c2_ corresponds to a critical current density *J*_c_ = 0.075*J*_dp_ [Fig. 7a] and field *B* = 0.5*H*_c2_ to *J*_c_ = 0.026*J*_dp_ [Fig. 7b]. On the exit side, the current density *J*_*x*_(*W*) reaches the depairing current density *J*_dp_.

## Discussion and Conclusions

In this article we studied the interplay of surface potential barrier and bulk pinning centres in mesoscopic superconducting strips, where both pinning mechanisms are relevant. Figure 2 suggests that the critical current reaches saturation at *W* ~ 64*ξ* in a clean strip, meaning that the effect of the surface barriers on *I*_c_ starts to decrease above that width and bulk defects become the dominant pinning mechanism. Since non-superconducting defects are detrimental for the Bean-Livingston barrier, we studied the general case of a non-homogeneous defect distribution across the width of the strip to be able to take advantage of both mechanisms. In particular, we assumed a linear modulation of the defect concentration near both edges of the strip. This allowed us to quantify the suppression of the surface barrier by defects in the vicinity of the strip edges by studying the vortex and supercurrent distribution in these regions.

Table 1 summarizes the results for our benchmark system — a strip of width *W* = 64*ξ* in a magnetic field *B* = 0.1*H*_c2_. The clean strip has a critical current density of ${J}_{{\rm{c}}}^{{\rm{clean}}}=0.081{J}_{{\rm{dp}}}$. For increasing strip width, the critical current density decreases as ~*W*^−1^, see Fig. 2, while approaching *J*_dp_ in the limit of very narrow clean strips with $W\lesssim \xi$. However, any defects or imperfections at the edges will significantly reduce these values. Adding random, but homogeneously distributed defects to the benchmark system increases *J*_c_ by 35% in the best case, which implies that the bulk pinning is more relevant than the suppression of the surface barrier for *B* = 0.1*H*_c2_ and *W* = 64*ξ*. This maximum bulk critical current at *B* = 0.1 is reached for a volume fraction occupied by defects of *f* = 0.2 and for defects with diameter *d* = 3*ξ*^39^. Increasing the width of these uniformly disordered strips, the effect from the edges become negligible and bulk pinning will be dominant, resulting in the critical current density approaching the one of an infinite 2D film (${J}_{{\rm{c}}}^{{\rm{2D}},{\rm{uniform}}}=0.104{J}_{{\rm{dp}}}$, i.e. comparable to ${J}_{{\rm{c}}}^{{\rm{uniform}}}$). Homogeneous defect distributions in narrower strips result in a noticeable suppression of the edge barrier, thus decreasing the critical current density (for $W\,\searrow \,d$ it is clear that *J*_c_ → 0).

**Table 1:** Critical currents in a strip of width *W* = 64*ξ* at magnetic field *B* = 0.1*H*_c2_ for different defect distributions: clean strip without defects, uniform concentration of defects, optimized concentration of defects in the bulk and near the edges, reduced defect concentration at both edges [*l*_in_, *l*_out_ > 0 in Eq. (5)], and reduced defect concentration at the vortex entrance and exit.

Type	*f*	*l*_in_	*l*_out_	*J*_c_	cf. clean	
clean	0.0	—	—	0.081*J*_dp_	100%	Fig. 2
uniform	0.2	0	0	0.108*J*_dp_	135%	Fig. 3
optimized	0.2	9*ξ*	31*ξ*	0.142*J*_dp_	178%	Eq. (4)
both	0.2	10*ξ*	30*ξ*	0.140*J*_dp_	175%	Fig. 4
in	0.2	10*ξ*	0	0.118*J*_dp_	148%	Fig. 5
out	0.2	0	30*ξ*	0.131*J*_dp_	164%	Fig. 6

The defects are circular with diameter *d* = 3*ξ*.

In order to extract more detailed information about the suppression of the Bean-Livingston barrier, we introduced linear defect modulations near the edges. Studying first the vortex entrance and exit edges independently, we found that defects have an asymmetric effect on either side of the strip. A linear increase of the defect density at the entrance edge over 10*ξ* increases the critical current density by another 9% compared to the uniform case. A density decrease at the exit edge over 30*ξ* adds 21% to *J*_c_ compared to the uniform distribution. Therefore, the exit side is more sensitive to the contamination by defects located at some distance to the surface.

Next, we studied non-uniform modulations near both edges, defined by Eq. (5). Subsequent optimization over its parameters *f*, *l*_in_, and *l*_out_ leads to ${J}_{{\rm{c}}}^{{\rm{opt}}}=0.142{J}_{{\rm{dp}}}$, which is 31% more than for the uniform density with optimal values *f* ^opt^ = 0.2, ${l}_{{\rm{in}}}^{{\rm{opt}}}=9\xi$, ${l}_{{\rm{out}}}^{{\rm{opt}}}=31\xi$. Compared to the clean strip this is a *J*_c_-increase of 78%. We note that the effects from both sides of the strip add up independently for our relatively wide strip of *W* = 64*ξ*. One can expect that those optimal values for *l*_in_ and *l*_out_ remain independent of *W* for wider strips, while their overall influence on *J*_c_ diminishes with increasing *W* as the edges are local. Important to note is, that the mesoscopic strip under consideration with non-uniform distribution of defects has a larger critical current density than a homogeneously disordered 2D film.

**Figure 8:**
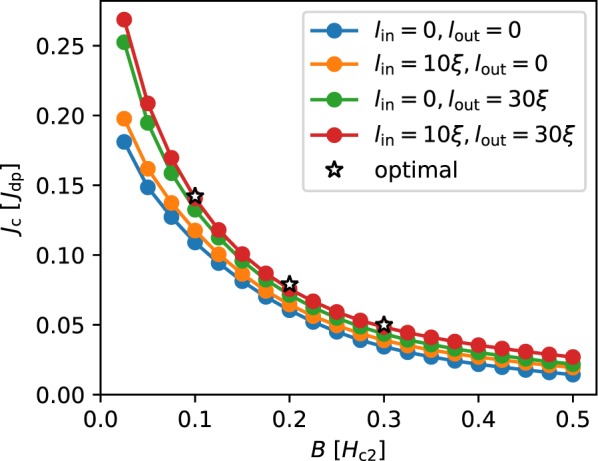
The critical current as a function of the external magnetic field for uniform distribution with *f* = 0.2 (blue), ‘in’ *l*_in_ = 10*ξ* (orange) and ‘out’ *l*_out_ = 30*ξ* (green) configurations, and ‘in’ + ‘out’ configuration with fixed *l*_in_ = 10*ξ* and *l*_out_ = 30*ξ*. The latter configuration is close to the configuration (empty stars) having maximal possible *J*_c_ for *B* = 0.1*H*_c2_, 0.2*H*_c2_, and 0.3*H*_c2_.

Finally, we studied the field dependence of the critical current for our *W* = 64*ξ* system, shown in Fig. 8. One clearly sees that the system with non-uniform defect distribution at both edge has the highest critical current density over a wide range of fields. Furthermore, as mentioned above, the system becomes less sensitive to the width of the linearly modulated edge regions as the optimal *J*_c_ value for *B* = 0.2*H*_c2_ and 0.3*H*_c2_ (indicated by stars) are almost sitting on top the field dependence of the system optimized for *B* = 0.1*H*_c2_ (red curve). Again, the homogeneously disordered system with optimal defect concentration has a lower *J*_c_ due to the suppression of the surface barrier. We can compare this result to the study of the interplay of bulk disorder and edge pinning presented in ref.^41^. In this work, the authors determined the field and pinning strength dependence of the critical current in a homogeneously disordered strip. Although the pinning strength (and therefore the bulk *J*_c_) was introduced as a phenomenological parameter, interestingly even in this case the authors found that the effect of the surface barrier and bulk pinning is not additive.

Overall, a non-homogeneous defect density modulation can significantly improve the critical current density in mesoscopic superconducting strips to higher values than those reached in 2D films.
